# Investigating the Antimicrobial Activity of Vancomycin-Loaded Soy Protein Nanoparticles

**DOI:** 10.1155/2022/5709999

**Published:** 2022-06-29

**Authors:** Hadi Zare-Zardini, Hossein Soltaninejad, Adel Ghorani-Azam, Mohammad Javad Forouzani-Moghaddam, Sima Mozafri, Zohreh Akhoundi-Meybodi, Farzad Ferdosian, Fatemeh Jabinian

**Affiliations:** ^1^Department of Biomedical Engineering, Meybod University, Meybod, Iran; ^2^Hematology and Oncology Research Center, Shahid Sadoughi University of Medical Sciences, Yazd, Iran; ^3^Medical Nanotechnology & Tissue Engineering Research Center, Yazd Reproductive Sciences Institute, Shahid Sadoughi University of Medical Sciences, Yazd, Iran; ^4^Faculty of Interdisciplinary Science and Technology, Tarbiat Modares University, Tehran, Iran; ^5^Department of Forensic Medicine and Toxicology, School of Medicine, Urmia University of Medical Sciences, Urmia, Iran; ^6^School of Paramedical, Iran University of Medical Sciences, Tehran, Iran; ^7^Department of Operating Room and Anesthesiology, School of Allied Medical Sciences, Tehran, Iran

## Abstract

Developing targeted and slow-release antibiotic delivery systems can effectively reduce drug overdose and side effects. This study aimed to investigate the antimicrobial activity of vancomycin-loaded soy protein nanoparticles (vancomycin-SPNs). For the preparation of SPNs, the desolvation method was applied in different concentrations of vancomycin and soy protein (15:5, 10:15, 6:20, 8:25, and 10:30 of vancomycin:soy protein). Scanning electron microscope (SEM), transmission electron microscopy (TEM), dynamic light scattering (DLS), and FTIR were used for nanoparticle characterization. Antibacterial activity was evaluated by the radial diffusion assay (RDA) and absorbance methods. Proper synthesis was demonstrated by characterization. The best drug loading (% entrapment efficiency = 90.2%), the fastest release rate (% release = 88.2%), and the best antibacterial activity were observed in ratio 10:30 of vancomycin:SPNs. Results showed that SPNs are a potent delivery system for antibiotic loading and slow release to control antibiotic use.

## 1. Introduction

Developing various antibiotics led to the promising treatment of bacterial infectious diseases [1]. Antibiotic resistance is the main problem of antibiotic use. Higher costs, prolonged hospitalization, and increased mortality can occur to antibiotic resistance [2]. Different strategies can be used to reduce antibiotic resistance [3]. Slow-release formulations can improve the therapeutical index of antibiotics and reduce antibiotic resistance [4, 5]. Nanostructures have potent properties for the slow and targeted drug release at an appropriate rate and the target site [6, 7]. Nanoengineered delivery systems can enhance antibiotic therapy efficiency by reducing limitations associated with antibiotic drugs [8, 9]. Protein-based nanostructures are suitable compounds for drug delivery systems, especially antibiotic delivery systems [10]. Biocompatibility, biodegradability, and low toxicity are the most important properties of these nanoparticles [11]. Among proteins, soy protein is one of the exciting candidates for the preparation of nanoparticle delivery systems due to its biodegradability, biocompatibility, low toxicity, low immunogenicity, and symmetrical shape [12, 13]. These nanoparticles can be used for encapsulation of various drugs [14]. So, the aim of this study is the synthesis, characterization, and antibacterial evaluation of vancomycin-containing soy protein nanoparticles (vancomycin-SPNs) as a new antimicrobial agent.

## 2. Materials and Methods

### 2.1. Nanoparticle Synthesis

For protein achievement, 10 g of soy proteins were precipitated by an ammonium sulfate gradient (0–85%) [15]. The desolvation method was used for the SPNs preparation. In this method, protein solution (with different concentrations of 1–15 mg/ml) was mixed with varying ethanol concentrations (0 to 80%) for 20 min on a stirrer at room temperature. Glutaraldehyde (0–95%) was added to a mixed mentioned solution as a stoichiometric cross-linker and incubated at room temperature. The solution was diluted by adding ethanol. Rotary evaporation was used for ethanol removal and replacement with deionized water. The acquired solution was centrifuged for 20 min. The supernatant was stored at 4°C [16].

### 2.2. Vancomycin Loading

**Figure 1:**
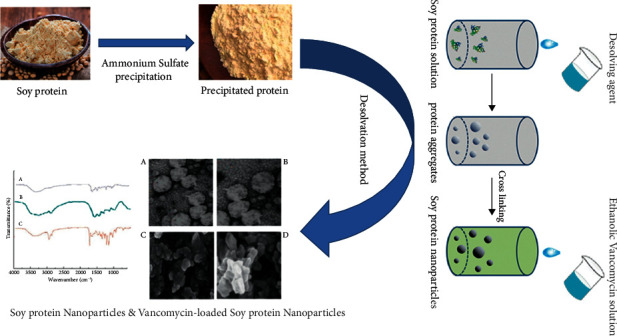
Schematic diagram of synthesis procedure by desolvation method.

For encapsulation of vancomycin-SPNs, ethanolic solution of vancomycin (EXIR Company, Iran) was added to the nanoparticle solution. Different concentrations of vancomycin and nanoparticles were used for this stage. After incubation at room temperature, centrifugation was applied for 20 min and the supernatant was stored at 4°C [10]. The synthesis procedure is shown in Figure 1.

### 2.3. Nanoparticle Characterization

Dynamic laser scattering (DLS) (SZ-100Z2, Horiba Company, Japan) and Dynamic Scattering software were used to evaluate nanoparticle size. Morphology assessment was performed by scanning electron microscopy (SEM) (Philips XI30, Philips Company, Netherlands) and transmission electron microscopy (TEM) (HT7800, Hitachi Company, Japan). Evaluation of functional groups was performed by the FTIR (Nicolet iS50, Thermo scientific Company, US) method.

### 2.4. Drug Loading and Release Evaluation

A standard curve was prepared by different concentrations of vancomycin and absorbance at 280 nm. Drug loading assessment was performed on PBS-diluted supernatant after centrifugation of 10 ml of the drug solution and the nanocarriers at 6,000 rpm for 20 minutes [17].

Entrapment efficiency percentage (percentage of drug that is successfully entrapped into nanoparticles = % EE) was calculated based on

(1) $$\begin{array}{c} \text{\%EE}=\left[\frac{\left(\text{Drug added}-\text{unentrapped drug}\right)}{\text{Drug added}}\right]\times 100\text{.} \end{array}$$

Loading capacity (percentage of the nanoparticles weight is composed of the drug = % LC) was calculated based on

(2) $$\begin{array}{c} \text{\%LC}=\left[\frac{\text{Entrapped Drug}}{\text{Nanoparticles weight}}\right]\times 100\text{.} \end{array}$$

Drug release evaluation was done by dialysis bag. Ten mg of lyophilized nanostructure was dissolved in PBS buffer (pH = 7.4) and poured into the dialysis bag. At different time intervals, 5 ml of the solution was removed and replaced with 5 ml of fresh buffer. The absorbance of the samples at each step was read at 280 nm [17].

### 2.5. Antimicrobial Activity Assessment

The radial diffusion assay (RDA) and absorbance-based methods were used for antimicrobial activity evaluation [18–20]. This test was performed on *Staphylococcus aureus* ATCC 25923 and *Streptococcus pyogenes* ATCC 19615 (Tamad Company, Iran). In the RDA method, after bacterial culture in TSA medium and solidification, six wells were created on medium for injection of five prepared nanostructures (15:5, 10:15, 6:20, 8:25, and 10:30 of vancomycin:soy protein) and one as control. The plates were incubated for 18 h at 37°C. The antimicrobial effects appear as an inhibition zone around the wells. Vancomycin and phosphate-buffered saline (PBS) were used as positive and negative controls.

In the adsorption method, the bacteria were cultures in 96-well microplates. 180 µl of the bacteria-containing medium was mixed with 20 µl of prepared nanostructure solutions in each well. In the control well, 180 µl of the bacteria-containing medium was mixed with 20 µl of PBS buffer. The microplate was incubated for 18 h at 37°C. Then, the ELISA reader evaluated bacterial growth by absorbance at 630 nm. The minimum concentration of nanostructures that have stopped bacterial growth is considered minimal inhibitory concentration (MIC).

## 3. Results

### 3.1. Characterization Results

The process of synthesis of vancomycin-SPNs was successfully performed by the desolvation method. FTIR, TEM, and SEM methods were used for the characterization of designed nanostructures. Glutaraldehyde was used as a linker. This linker is attached to SPNs amine groups of *R* in lysine residues. After synthesis, electrostatic bonding was formed between vancomycin's positive charge and the nanocarrier's negative charge.

### 3.2. FTIR Result

**Figure 2:**
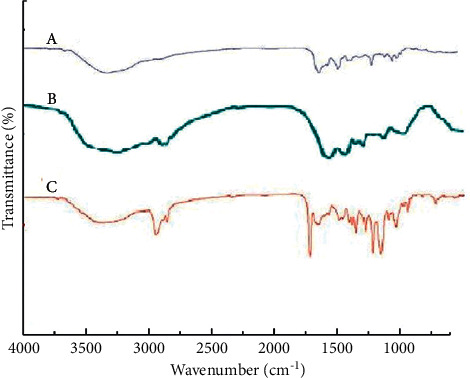
FTIR spectra of designed nanostructures. (a) Vancomycin, (b) soy protein nanoparticle (SPNs), and (c) vancomycin-loaded soy protein nanoparticle (vancomycin-SPNs). Characteristic changes in intensity and displacement of the index peaks in the vancomycin-SPNs compared to the free drug and the drug-free SPNs.

The result of the FTIR analysis was summarized in Figure 2. In vancomycin and vancomycin-SPNs, characteristic peaks at 1652, 1588, and 1506 cm^−1^ belong to amide I, amid II, and the aromatic group of vancomycin, respectively. These observed peaks showed the presence of vancomycin in vancomycin-loaded soy protein nanoparticles. In drug-free SPNs and vancomycin-SPNs, characteristic peaks at 3294, 1655, and 1535 cm^−1^ belong to O–H and N–H, amide I, and amid II, respectively. The change in intensity and displacement of the index peaks in the vancomycin-SPNs compared to the free drug and the drug-free SPNs showed the accuracy of functionalization.

### 3.3. SEM and TEM Characterization

**Figure 3:**
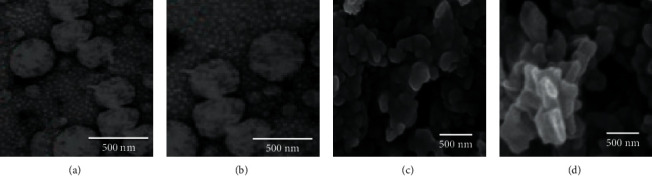
TEM and SEM image of synthesized nanostructures. (a) TEM image of soy protein nanoparticles (SPNs), (b) TEM image of vancomycin-loaded soy protein nanoparticles (vancomycin-SPNs), (c) SEM image of SPNs, and (d) SEM image of vancomycin-SPNs. Spherical and oval shapes of the drug-free SPNs and vancomycin-SPNs in TEM images. Similar shapes for soy protein nanoparticles and vancomycin-loaded soy protein nanoparticles in SEM images.

TEM showed the spherical shape of the drug-free SPNs and vancomycin-SPNs (Figure 3(a) and 3(b)). In some drug-containing nanoparticles, deformation from spherical to the oval was observed. However, this deformation is not very noticeable. SEM imaged showed that drug loading did not significantly change the shape of the nanoparticles (Figure 3(c) and 3(d)). The only visible difference is the elongation of the nanoparticle surface, especially at the corners, which causes the nanoparticle to deviate slightly from the spherical state. This change is more evident in some particles, and others are hardly comparable to drug-free nanoparticles. These figures also indicated that the drug-free SPNs are smaller than the vancomycin-SPNs, to a small extent.

The size and zeta potentials were 385.3 nm and −34.2 mV for SPNs and 412.84 nm and −28.9 for vancomycin-SPNs (Table 1).

### 3.4. Investigation of Drug Loading and Release

**Figure 4:**
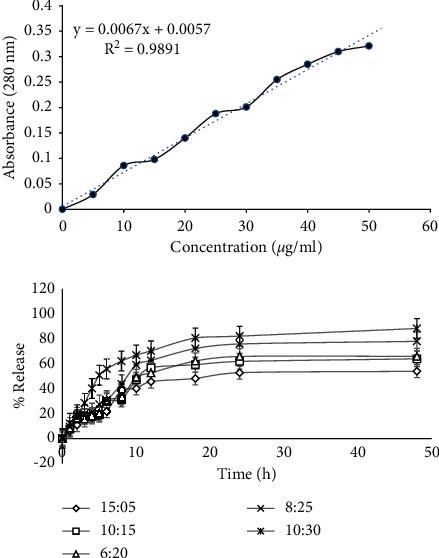
Standard curve of vancomycin based on absorbance at 280 nm and drug release in different concentrations (mg/ml) of vancomycin:soy protein nanoparticles (vancomycin:SPNs). 15:5, 10:15, 6:20, 8:25, and 10:30 of vancomycin:SPNs.

The best drug loading occurred in the ratio of 10:30 vancomycin/soy protein nanoparticles (% EE = 90.2% and % LC = 41.5%). The relationship between drug loading and nanoparticle concentration is linear, meaning that the higher the nanoparticle concentration, the higher the drug loading rate. Drug release evaluation showed that the ratio of 10:30 vancomycin/soy protein nanoparticles simultaneously had the highest drug release percentage compared to other ratios (Figure 4). According to data, for the ratio of 10:30 vancomycin/soy protein nanoparticles, the maximum drug release during 48 hours was 88.2%.

### 3.5. Antibacterial Activity

**Figure 5:**
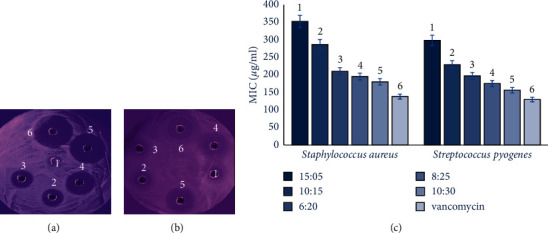
Antimicrobial activity of vancomycin and vancomycin/soy protein nanoparticles (vancomycin-SPNs) based on RDA test on *Staphylococcus aureus* (a) and *Streptococcus pyogenes* (b). Minimum inhibitory concentration (MIC) values for vancomycin and vancomycin-SPNs (c). (1) 15:05 (mg/ml of vancomycin:SPNs); (2) 10:15 (mg/ml of vancomycin:SPNs); (3) 6:20 (mg/ml of vancomycin:SPNs); (4) 8:25 (mg/ml of vancomycin:SPNs); (5) 10:30 (mg/ml of vancomycin:SPNs), and (6) vancomycin.

For assessment of antibacterial activity, RDA and absorbance methods were used. In the RDA method, the best antimicrobial activity in all studies bacteria belonged to 10:30 of vancomycin:soy protein. Antimicrobial activity increased with decreasing the ratio of vancomycin/nanoparticle. Naked vancomycin showed the highest antibacterial activity (Figure 5). The results of MIC determination were similar to RDA data. As shown in Figure 5, bacteria were sensitive to vancomycin and different concentrations of vancomycin/nanoparticle. Among vancomycin/nanoparticles, identical to RDA data, the ratio of 10:30 showed higher antibacterial activity than other ratios. This activity was lower than naked vancomycin. These data showed that more soy proteins lead to more significant antimicrobial activity.

## 4. Discussion

One of the biggest health threats is infectious disease. This disease is known as one of the most common causes of mortality in hospitals. The development of treatment strategies can effectively reduce its related mortality [20]. Antibiotic treatment is commonly used for bacterial infections [21–23]. Although antibiotic therapy is a standard treatment for infectious diseases, the increasing use of these compounds has led to the development of antibiotic resistance [23]. One of the best ways to reduce antibiotic resistance is drug delivery systems. This strategy improves drug absorption, allows targeted antibiotic delivery, improves their tissue and biofilm penetration, and reduces side effects [24]. This study prepared a delivery system based on SPNs for vancomycin. Based on the literature review, protein-based nanoparticles have been considered exciting drug delivery systems due to their long gastrointestinal residence, high tissue penetration, amphipathic properties, low toxicity on mammalian cells, biodegradability, biocompatibility, and nonantigenic properties [14, 16, 25]. Protein nanoparticles can be generated by various proteins such as fibroins, albumin, gelatin, gliadine, legumin, lipoprotein, ferritin, and soy proteins [26]. Soy proteins are one of the most abundant plant proteins. These proteins have been extensively used as drug delivery system [27]. We used soy proteins for preparation nanoparticles ad drug delivery system. There are different methods for preparing various forms of soy protein structures (microspheres, hydrogels, polymer blends, and nanoparticles) [12]. The desolvation method is a powerful strategy for synthesizing soy protein nanoparticles. In this method, nanoparticles are obtained when a desolvating agent is added to an aqueous protein solution under stirring to dehydrate the protein, resulting in a conformational change from stretched to coil conformation [28, 29]. In this study, this method was also used for SPNs preparation [30]. The ratio of protein to ethanol concentration significantly affects the synthesis process [31]. In our study, the best nanoparticles were formed in 5 mg/ml of soy proteins and 40% of the ethanolic solution. The mean size of SPNs in this condition was 385.3 nm. The zeta potential of SPNs was −34.2 mV. The use of ethanolic solution leads to the induction of negative charge on the surface of acquired nanoparticles due to ionization of the charged groups in soy protein molecules [32]. The net charge of SPNs increases along with the enhancement of ethanol concentration. Our study showed similar data. The sizes and zeta potentials of free drug and drug-loaded SPNs are different. However, the net charge of both of them is negative. Based on the results, after vancomycin functionalization, the net charge of particles decreases and their size increases (size = 412.84 nm and zeta potential = −28.9 for 10:30 vancomycin/SPNs). The reduction of charge and increase of size are directly related to drug concentration. The smaller size and higher charges was observed in 10:30 vancomycin/SPNs. If the proper vancomycin/SPNs selected, the effect of vancomycin on size will be decreased. The high surface area/volume ratio helps drug loading capacity on nanostructures. Our study indicated suitable drug loading on SPNs in all proportions of vancomycin/SPNs. Surface area enhancement leads to the enchantment of functional group availability, especially active groups in electrostatic interactions [33, 34]. Thus, drug concentrations cannot have considerable effects on drug loading capacity. This capacity is mainly related to nanoparticle contents. So, a different %EE is due to a change in the ratio of SPNs. There are similar patterns in drug release in all prepared nanostructures. In 48 h, the lowest drug release was observed in the proportion of 10:30 vancomycin:SPNs. Based on similar studies, spherical nanoparticles have more efficient action than other shapes of nanostructures such as nanotubes, graphene, and hydrogel [29, 35, 36]. SPNs in higher concentrations (10:30) can maintain antibiotics for a long time and control drug release more regularly. After examining the release profile of the drug at different times, the results indicate that the synthesized nanosystem is a slow-release system. For investigation of vancomycin release kinetics, the cumulative release chart was examined based on four models: zero, logarithmic, and Higuchi (Table 2). The results showed that the Higuchi equation is more suitable for fitting experimental data. Like other reported articles, free antibiotics have higher antimicrobial activity than antibiotic-loaded nanostructures. Slower drug release and decreased solubility are two critical reasons for the lower antimicrobial activity of antibiotic-loaded SPNs [31, 32, 37, 38]. Slow-release drugs can control antibiotic toxicity in high concentrations. On the other hand, functionalizing SPNs surface by cellular marker can be effective in targeted antibiotic delivery. In comparison with free drug, loaded drug in SPNs has lower solubility. This low solubility can help reach higher drug concentrations at the infection site. Encapsulation of vancomycin in SPNs can also lead to a reduction of its toxicity. The best antibacterial activity was observed in a ratio of 10:30 vancomycin:SPNs. Other factors, including slow-release, control release, spherical shape, and drug loading are ideal for this ratio. In in vivo conditions, higher concentrations of trapped vancomycin in nanocarriers will reach the site of infection compared with free vancomycin, as high vancomycin solubility also accelerates vascular excretion. On the other hand, due to the toxicity of vancomycin, its addition to carriers such as SPNs can effectively reduce the drug's toxic effects, including renal toxicity.

## 5. Conclusion

Based on results, SPNs with a defined ratio (10:30 vancomycin:SPNs) can be used as a delivery system for vancomycin. The prepared vancomycin:SPNs showed low toxicity, slow and control release, spherical shape, best drug loading, and potent antibacterial activity. The concentrations of protein and desolvating agent (ethanol) can affect the size of prepared SPNs. The ideal ethanol concentration was 40% for preparing 10:30 vancomycin:SPNs. According to the results, SPNs can be used as a slow and controlled release drug delivery system for vancomycin, especially in the ratio of 10:30 vancomycin:SPNs.

## Figures and Tables

**Table 1:** Characteristics of size and zeta potential of free SPNs and vancomycin-SPNs.

Nanoparticles	Size (nm)	Zeta potential (mV)
SPNs	385.3	−34.2
Vancomycin-SPNs (10/30)	412.84	−28.9

**Table 2:** Drug release based on the zero, 1, logarithmic, and Higuchi equation of SPNs with ratios of 10/30 (vancomycin/SPNs).

Equation degree	*R* ^2^	Slope
0	0.7218	0.0162
1	0.7812	0.0031
Logarithmic graph	0.8521	0.0075
Higuchi	0.9867	0.651

## Data Availability

All the data are available in the manuscript.
